# Impact of semi-solid formulations on skin penetration of iron oxide nanoparticles

**DOI:** 10.1186/s12951-017-0249-6

**Published:** 2017-02-17

**Authors:** Umberto M. Musazzi, Benedetta Santini, Francesca Selmin, Valentina Marini, Fabio Corsi, Raffaele Allevi, Anna M. Ferretti, Davide Prosperi, Francesco Cilurzo, Miriam Colombo, Paola Minghetti

**Affiliations:** 1 Department of Pharmaceutilcal Sciences, Università degli Studi di Milano, via G. Colombo, 71, 20133 Milan, Italy; 2 Department of Biotechnology and Biosciences, Università degli Studi di Milano-Bicocca, Piazza della Scienza, 2, 20126 Milan, Italy; 3 Dipartimento di Scienze Biologiche e Cliniche, Università degli Studi di Milano, via G. B. Grassi, 74, 20157 Milan, Italy; 4 Lab. di Nanotecnologie, CNR-Istituto di Scienze e tecnologie Molecolari, via G. Fantoli 16/15, 20138 Milan, Italy

**Keywords:** Iron oxide nanoparticles, Polymer coating, Semi-solid preparation, Skin penetration, Nanoparticle stability

## Abstract

**Background:**

This work aimed to provide useful information on the incidence of the choice of formulation in semi-solid preparations of iron-oxide nanoparticles (IONs). The appropriate analytical methods to assess the IONs physical stability and the effect of the semi-solid preparations on IONs human skin penetration were discussed. The physical stability of IONs (D_h_ = 31 ± 4 nm; ζ = −65 ± 5 mV) loaded in five semi-solid preparations (0.3% w/v), namely Carbopol gel (CP), hydroxyethyl cellulose gel (HEC), carboxymethylcellulose gel (CMC), cetomacrogol cream (Cet) and cold cream was assessed by combining DLS and low-field pulsed NMR data. The in vitro penetration of IONs was studied using human epidermis or isolated stratum corneum (SC).

**Results:**

Reversible and irreversible IONs aggregates were evidenced only in HEC and CMC, respectively. IONs diffused massively through SC preferentially by an intercellular pathway, as assessed by transmission electron microscopy. The semi-solid preparations differently influenced the IONs penetration as compared to the aqueous suspension. Cet cream allowed the highest permeation and the lowest retained amount, while cold cream and CP favored the accumulation into the skin membrane.

**Conclusion:**

Basic cutaneous semi-solid preparations could be used to administer IONs without affecting their permeation profile if they maintained their physical stability over time. This property is better discriminated by low-field pulsed NMR measurements than the commonly used DLS measurements.

**Electronic supplementary material:**

The online version of this article (doi:10.1186/s12951-017-0249-6) contains supplementary material, which is available to authorized users.

## Background

Skin is one of the focuses for research in drug delivery with many drugs being evaluated for transdermal or dermal administration. However, penetration and retention of drugs into the epidermis is not a simple task. Indeed, the outermost layer of the skin, the stratum corneum (SC), is a barrier both to water transport out of the body and to inward chemical permeation. In addition to the physical barriers, the clearance of capillaries in the dermis and the cutaneous metabolism via local phase I and phase II metabolic enzymes can also reduce the local bioavailability of drugs [1]. Among the possible approaches to overcome these issues, nanocarriers represent a new opportunity to improve the treatment of loco-regional diseases. For instance, nanoparticle dispersions (e.g., vesicles) have been applied to transcutaneous delivery of drugs, with some of them being commercialized and many more under clinical assessment [2].

Metal-based nanoparticles provide new perspectives on particle absorption into/through the skin, since their particle size and shape could be tailored to favor the penetration of SC via the intercellular route [3–5]. Nevertheless, most of the studies hinge on the potential toxicity effect of the nanoparticles through the skin and the mechanism related to the interaction between nanoparticles and skin layers [6, 7]. Multifunctional colloidal nanoparticles have been designed to improve the delivery of conventional drugs, peptides, vaccines or genes across biological barriers, including blood brain barrier, dermal, transdermal and intraocular delivery [8]. Among the plethora of metal-based colloidal nanoparticles, the use of iron oxide nanoparticles (IONs) appears of particular interest since they have been already used in several medical areas, including therapeutics and diagnostics, and, therefore, their safety profiles are already known [9, 10]. In vitro transdermal studies demonstrated that the IONs bearing antitumor drugs were able to penetrate the skin assisted by an applied magnetic field, suggesting a potential of IONs as drug delivery system for transdermal therapy of skin cancer [11].

However, the actual broad applicability of IONs in the clinical practice remains controversial due to contradictory evidence on potential toxicity of colloidal nanoparticles. Recent studies suggested that appropriate surface coating with “bio-friendly” materials might improve the nanoparticle stability in biological environment, reducing the oxidative stress mediated toxicological effects as well as immune and carcinogenic effects and preserving the physiological processes [12, 13].

Intravenous administration of colloidal nanoparticles is still matter of debate. Intravenous and oral administrations of IONs formulations have been approved for clinical use by the Food and Drug Administration (FDA) [14]. However, most intravenously administered compounds approved by FDA have been withdrawn from the marketplace [15], with the exception of some therapeutics indicated for the treatment of iron deficiency anemia in adults [16]. IONs for oral administration remain on the market for use as an oral gastrointestinal contrast agent in MR imaging (i.e., Gastromark® and Ferumoxsil-containing products) [17]. For these reasons, the skin penetration of IONs has been gaining increasing interest in nanomedicine [9, 18, 19]. It has been suggested that IONs with a diameter smaller than 40 nm were able to penetrate the intercellular space among keratinocytes of the SC [18] and 10 nm-IONs were able to penetrate the human epidermis (HE), without reaching the dermal layer [20]. In addition, the IONs polymeric coating, which is required to obtain a colloidal solution stable both in water and in physiologic media, should be carefully selected since an isoelectric point similar to that of human skin would favor the formation of IONs clusters at the skin interface, preventing their permeation [20].

In a preliminary study, IONs coated by a poly-(isobutylene-alt-maleic anhydride) (PMA-IONs) were demonstrated to accumulate in ex vivo full-thickness human skin and in mice skin after topical application in vivo [21]. However, physicochemical attributes, such as size, surface charge, surface chemistry, and physical state of the nanoparticles are not the only critical determinants for their skin permeation. Indeed, the diffusion performance relies also on the physicochemical properties and composition of the semi-solid formulation required to obtain a suitable cutaneous product. Such a vehicle includes other excipients to afford the lubricity and spreading properties, the desired residence time on skin and the patient compliance. Although the stability of IONs, or more in general of nanoparticles, in the vehicle has been one of the major critical factors that could affect the effectiveness of their topical administration, very few data were reported in literature. In particular, no systematic studies have been carried out to investigate the influence of semi-solid formulations on the physicochemical features of IONs and on their permeability across the human skin. Therefore, to rationalize the selection of a suitable vehicle for PMA-IONs administration, this work aimed to investigate the impact of semi-solid formulations (i.e., hydrogels and o/w creams) on the physical stability of nanoparticles and on the in vitro penetration through HE.

## Results

### Formulation study of PMA-IONs semi-solid preparations

**Fig. 1:**
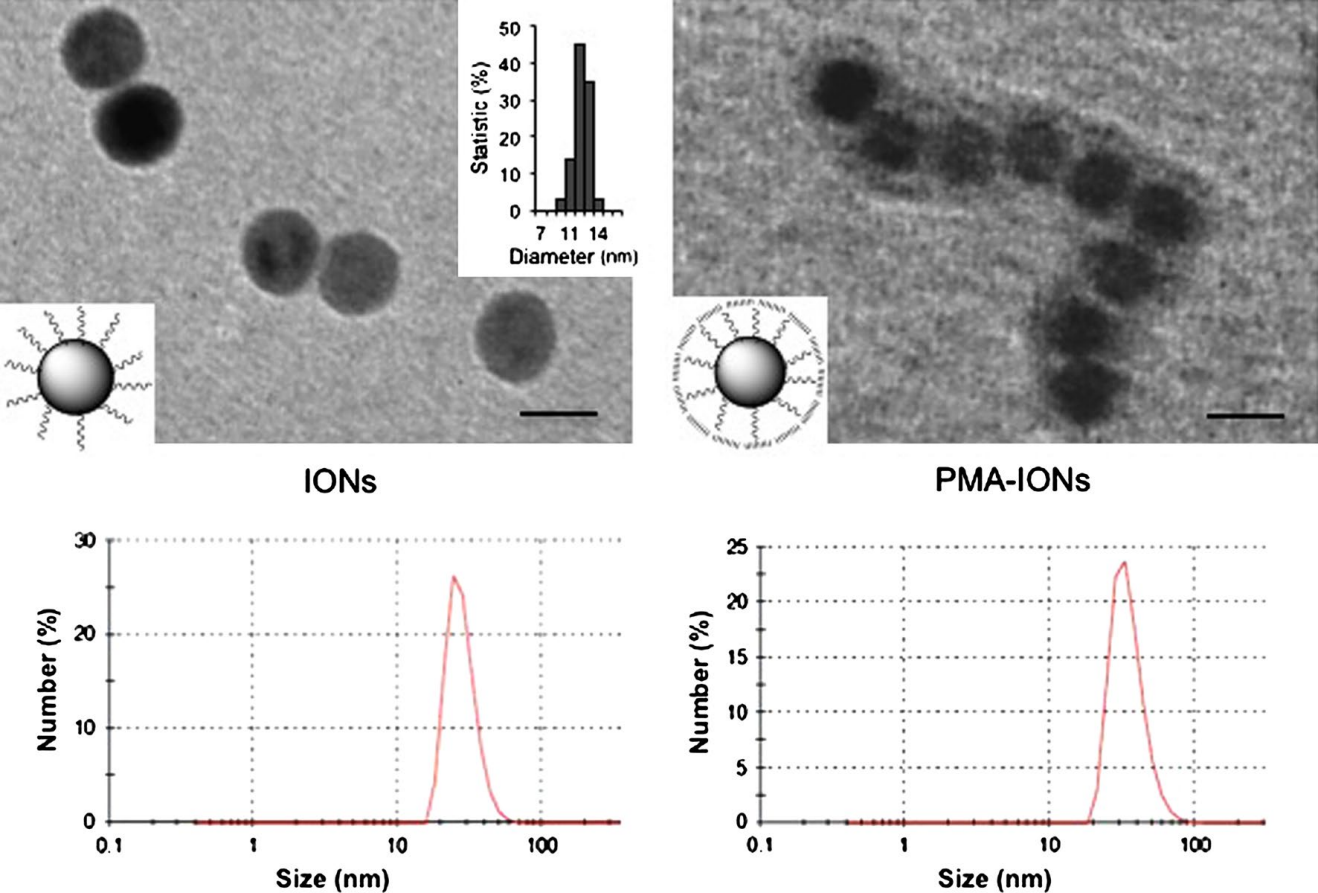
TEM images and dynamic light scattering (DLS) of iron oxide NPs (IONs) in organic solvent and water phase transfer using PMA amphiphilic polymer (PMA-IONs). *Scale bars* 10 nm

The protocol adopted for the production of PMA-IONs allowed to obtain monodisperse nanoparticles with a strongly negative surface charge (Fig. 1; Table 1). PMA-IONs were loaded in three hydrogels, made of hydroxyethyl cellulose (HEC), sodium carboxymethyl cellulose (CMC) and carbomer 974P (CP), and two hydrophilic creams, namely a cetomacrogol (Cet) cream and a cold cream.

**Table 1:** Physical characterisation of PMA-IONs and PMA-IONs-loaded hydrogels (mean ± SD, n = 3)

Formulation	D_h_ (nm)	PDI	ξ (mV)	pH
PMA-IONs	31 ± 4	0.15 ± 0.04	−64.80 ± 4.91	7.0
HEC	29 ± 15	0.44 ± 0.05	−42.17 ± 1.68	5.0
CMC	47 ± 7	0.54 ± 0.03	−79.77 ± 2.00	6.5
CP	52 ± 4	0.29 ± 0.04	−72.83 ± 5.25	6.0

*HEC* hydroxyethyl cellulose, *CMC* sodium carboxymethyl cellulose, *CP* carbomer 974P

The physical stability of PMA-IONs loaded into the semi-solid formulations was determined according to two different methods, namely low-field pulsed NMR and dynamic light scattering (DLS), using the physicochemical features of the aqueous suspension as reference. The former technique was selected because it could provide direct characterization of the semi-solid preparation, while the latter allowed to assess the possible formation of nanoparticle aggregates or change in size.

**Fig. 2:**
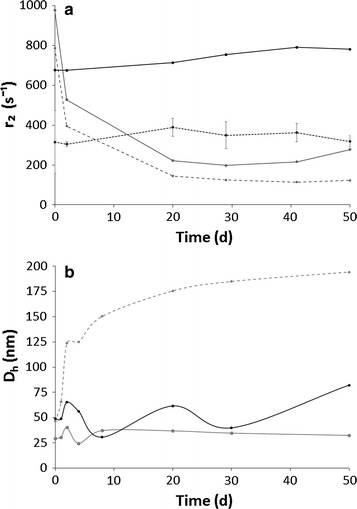
Variation of the *r*_2_ (**a**) and D_h_ values (**b**) of PMA-IONs loaded in hydrogels made of HEC (*grey*, *solid line*), CMC (*grey*, *dash line*) and CP (*black*, *solid line*) over time. The water suspension of PMA-IONs (*black*, *dash line*) was used as control

Low-field pulsed NMR analysis provided information on the magnetic properties of nanoparticles, which are influenced by deviation in structure, surface composition, concentration and mobility. Hence, the measurement of the *r*_2_ value of PMA-IONs was a sensitive parameter to characterize changes in the nanoparticles mobility in different media because its variation suggested the formation of interactions among the formulation components. As expected, the *r*_2_ value of the nanoparticles suspension in aqueous solution did not significantly change within 50 days of storage at 25 ± 2 °C, confirming the stability of PMA-IONs in water. A similar trend was evident analyzing nanoparticles dispersed in both o/w cream compositions (Additional file 1). Consequently, it might be assumed that neither aggregation nor alteration in size of iron oxide core occurred within this time window, as well as no modification of surface coating was detected over the time. The same trend was measured in the CP hydrogel. Conversely, in the case of HEC or CMC hydrogels, a sharp decrease of *r*_2_ values was noticed after one day of storage (Fig. 2a; Additional file 1). In particular, *r*_2_ value of CMC decreased due to PMA-IONs aggregation followed by a precipitation that became visually noticeable after 30 days.

Unlike low-field pulsed NMR technique, DLS measurements could not be performed directly on the semi-solid vehicles, but a prior sample dilution was required. Preliminarily, placebo hydrogels and o/w creams were analyzed by DLS to evaluate possible interferences. Adapting the protocol of sample preparation, DLS technique could be used only for analyzing hydrogels. On the contrary, it could not be exploited to verify the physical state of PMA-IONs incorporated into o/w creams because the dispersed oily phase and the formulation viscosity did not allow to obtain reproducible results, despite the dilution. As depicted in Fig. 2b, the DLS trends found for CMC and CP were almost superimposable during storage at 25 °C: PMA-IONs loaded in CMC hydrogel confirmed to be unstable, whereas the CP hydrogel appeared physically stable using both techniques. Contradictory results were found in the case HEC. Even if the low-field pulsed NMR evidenced instability of PMA-IONs in HEC hydrogel, no aggregates were noticed by DLS technique. These different patterns might be explained considering that the cellulose chains were able to promote the formation of reversible aggregates of PMA-IONs, which were not detectable by DLS due to the sample dilution. The formation of such reversible aggregates might be also in agreement with the very significant reduction of the absolute ξ values (Table 1), which did not undergo to significant variations over the considered time period (Additional file 1).

Increasing the storage temperature at 40 °C, the trends in DLS data were confirmed, although in case of IONs loaded in CMC hydrogel physical instability was observed after 4 days of storage (Additional file 1).

Due to the physical instability of PMA-IONs loaded in the CMC hydrogel, such vehicle was not considered worthy for further investigations.

### In vitro permeation studies

**Fig. 3:**
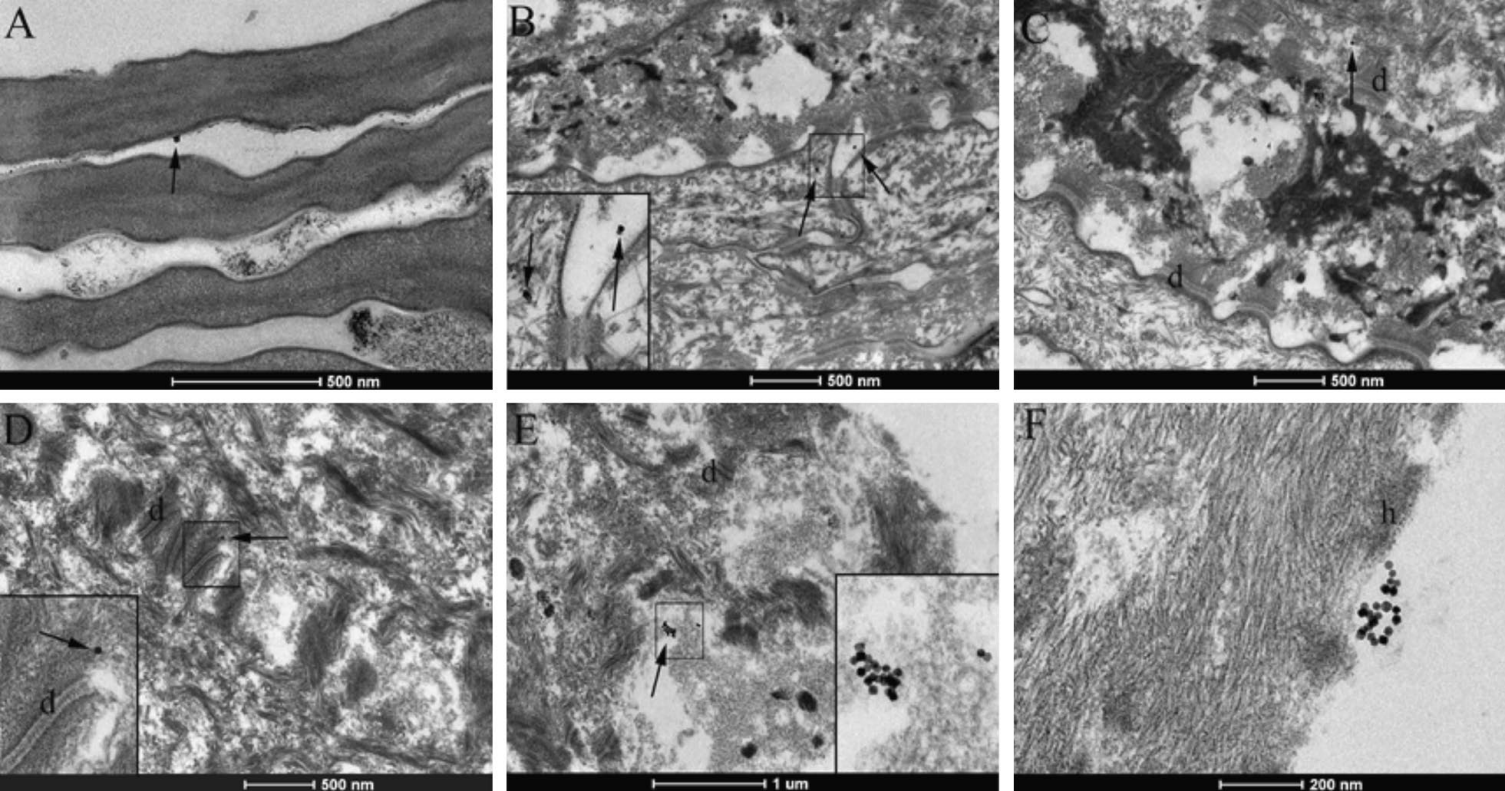
Transmission electron microscopy (TEM) images of skin samples treated with semisolid-formulations loaded by PMA-IONs. Nanoparticles (*arrows*) singularly or in cluster were found in stratum corneum (**A**), in stratum lucidum (**B**), in stratum granulosum (**C**), in stratum spinosum close to desmosomes (**D**), in stratum basale (**E**) and in proximity of desmosomes (**F**). *d* desmosomes, *h* hemidesmosomes

Transmission electron microscope (TEM) images showed the presence of nanoparticles both into the upper HE layers and near to the basal membrane of epidermis (Fig. 3). Indeed, after 24 h, PMA-IONs were found on the surface and close to the corneocytes, into the epidermis and near the basal membrane (Fig. 3). Figure 3F showed nanoparticles into a cell of the stratum lucidum inside the cytoplasm, while Fig. 3C showed nanoparticles close to desmosomes. Thus, even though PMA-IONs were present in the extracellular matrix, they showed also affinity for the proteins of the intercellular junctions.

**Fig. 4:**
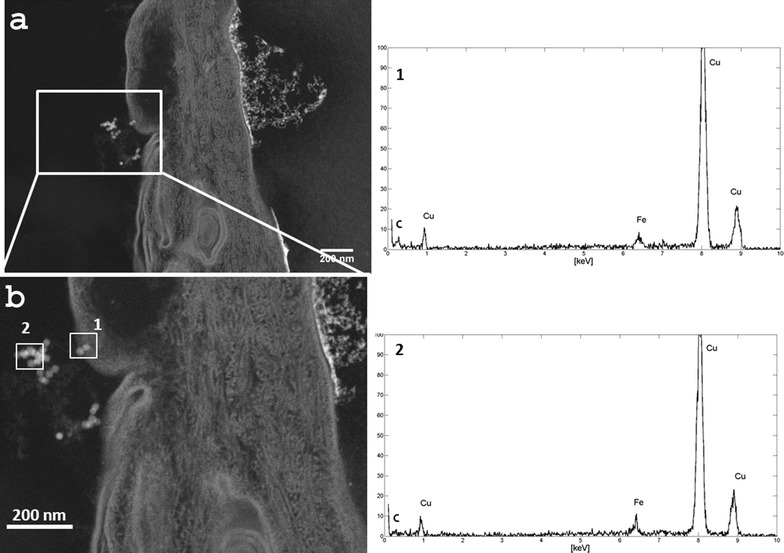
*On the left* **a** STEM image of HE tissue with some IONs; **b** enlargement of image **a**: the *white squares* highlight the areas of the EDX spectra. *On the right* the EDX spectra taken from the nanoparticles *highlighted* in **b**. The copper signals come from the TEM grid

In order to identify correctly the nature of that nanoparticles revealed by the conventional TEM in the HE sample, the STEM/EDX analysis was also carried out [22]. The STEM images were collected in different sections of the same sample previously analyzed by conventional TEM. Also STEM showed images with high degree of details. In particular, STEM micrographs show nanostructures close to the stratum corneum brighter than the other elements. These white regular spots are indicated by the white squares 1 and 2 in Fig. 4 and another brighter area (Additional file 1). The higher brilliance of these elements suggests that they are more electron dense and heavy than the other parts of the sample, without providing information on their chemical composition. So, the EDX analysis was performed on these white spots pointing the electron beam directly on them to determine their elemental composition. The spectra reported on the right side of STEM micrographs evidenced the presence of the iron signals only in squares 1 and 2 of Fig. 4, confirming that the nanoparticles are IONs. The EDX spectra revealed also the presence of a weak C-signal and a strong Cu-signal, which was attributed to the epidermis and TEM grid used to support the analyzed section, respectively.

This result was in agreement with the in vitro permeation study. Indeed, after 24 h, the retained amounts of PMA-IONs in SC and VE, normalized by the weight of the epidermal layers, were 234 ± 101 and 308 ± 101 ng/mg, respectively (Table 2).

**Table 2:** Results of permeation (Q_24_) and retention (Q_ret_) studies of aqueous suspensions and semisolid preparations containing 3 mg/mL of PMA-IONs

Formulation	Q_ret_		Q_24_		Q_ret_/Q_24,HE_
	A (μg/cm^2^)	N	A (μg/cm^2^)	N	
Water suspension	2.91 ± 1.04	1.98 ± 0.70	4.35 ± 0.74	2.30 ± 0.14	0.67
HEC hydrogel	1.73 ± 0.34	1.18 ± 0.23	3.48 ± 0.30	1.84 ± 0.16	0.50
CP hydrogel	4.70 ± 0.25	3.19 ± 0.17	7.22 ± 2.40	3.83 ± 1.27	0.65
Cet cream	1.98 ± 0.57	1.34 ± 0.39	10.51 ± 2.46	5.57 ± 1.30	0.19
Cold cream	5.30 ± 0.74	3.60 ± 0.50	8.05 ± 4.50	4.26 ± 2.39	0.66
Blank	1.47 ± 0.08	1.00 ± 0.05	1.89 ± 0.26	1.00 ± 0.14	–

The data were reported as absolute values (A) or as normalize values with respect to blank (N) (mean ± SD; n = 3)

*HEC* hydroxyethyl cellulose, *CMC* sodium carboxymethyl cellulose, *CP* carbomer 974P, *Cet* cetomacrogol

In addition, the iron amount permeated through HE after exposure to PMA-IONs for 24 h (p < 0.01) was doubled with respect to the pre-exposure HE, which was reported to contain traces of iron (Table 2) related to the intense mitotic activity of VE [23]. This finding suggested that PMA-IONs were able to permeate the SC barrier and to concentrate into the VE.

Membranes based on regenerated keratins (RKM) [24] were used to model the tendency of nanoparticles to diffuse through intracellular pathway of SC, because the lower the permeation through RKM, the higher the affinity of nanoparticles for the intracellular compartment of SC. The results showed that Q_24,RKM_ (6.4 ± 0.1 μg/cm^2^) was in accordance with the Q_24,HE_ values (Table 2).

On the other side, the Q_ret,RKM_ (28 ± 5 ng/mg) was significantly lower than the value obtained through SC (p value: 0.024). Hence, it may be assumed that weak interactions occurred between PMA-IONs and keratins and that PMA-IONs diffused preferentially through an intercellular pathway [25]. This hypothesis was also in agreement with the TEM microphotographs evidencing the presence of nanoparticles only in the interstitial spaces among keratinocytes (Fig. 3).

The components of the four semi-solid vehicles differently influenced the skin penetration of PMA-IONs. With the exception of the HEC hydrogel, all formulations significantly increased the permeated amount after 24 h, while only CP hydrogel and cold cream were able to increase also the amount of PMA-IONs retained into the HE (Table 2). Although the Q_ret_/Q_24,HE_ ratio of all the tested semi-solid formulations were similar to that of aqueous suspension (i.e., about 0.67), the permeated iron amount from the Cet cream was one order of magnitude higher than the relative retention data (Q_ret_/Q_24,HE_: 0.19), suggesting a higher tendency of PMA-IONs to permeate than to be retained into the HE when they were loaded in the Cet cream.

## Discussion

The permeation of nanoparticles through human skin is still matter of debate. Indeed, scientific evidence suggested that only those nanoparticles with size below the 6–7 nm limit were able to permeate the healthy skin through the lipidic trans-epidermal, whereas nanomaterials larger than 36 nm could be preferentially absorbed by the aqueous pores or trans-follicular routes [18].

PMA-IONs proposed in the current work exhibited a significant penetration through the human epidermis. Indeed, both TEM microscopy and permeation studies supported the evidence that PMA-IONs permeate significantly through the SC and accumulated into the VE. This behavior resulted dissimilar from that described for two types of 10 nm-IONs stabilized with a different coating [20]. In both cases, the nanosystems permeated the SC without reaching the VE in a massive amount. Hypothesizing that PMA-IONs permeate human skin following the same concentration-gradient mechanism of smaller chemical molecules, such incongruities may be explained on the basis of the differences in the experimental protocols, e.g. the type of membrane model. The experimental results of PMA-IONs were performed using HE, whereas full-thickness human skin has been used as membrane model by Baroli and co-authors. As discussed by Cross and Roberts, HE can model an in vivo “infinite dermal perfusion”, where the penetrated solute is fully removed from below the epidermal-dermal junction [26]. On the contrary, no clearance of solute from the dermis is expected using full thickness skin, suggesting that such membrane can be a model of in vivo “infinite dermal vasoconstriction”. In this context, the permeation profile of PMA-IONs may be increased by using an “infinite dermal perfusion” model with respect to other models of cutaneous barrier.

Besides such differences in the experimental protocols, the incongruities between experimental and published results may be also explained by a different tendency to distribute and diffuse through SC due to the different coating of IONs. The coated-IONs investigated by Baroli and co-authors were only stabilized in water by electrostatic interactions with sodium bis-(2-ethylhexyl) sulfosuccinate or tetramethylammonium hydroxide, whereas the use of PMA provided several advantages. Indeed, the amphiphilic property of PMA allowed to improve the colloidal stability of nanoparticles at physiological pH and their surface functionalization taking advantage of the presence of activated carboxylic groups on the surface. Another important advantage of the amphiphilic coating was that it could improve the IONs incorporation into a semi-solid formulation, their permeation through hydrophobic/hydrophilic biological environment and their penetration of SC by increasing their affinity for keratin or lipids present in the SC.

To make the topical administration of nanosystems clinically acceptable, they should be incorporated in semi-solid preparations, namely complex vehicles that potentially could affect their permeation performances. Exception made for HEC, the results demonstrated that all the tested semi-solid vehicles improved PMA-IONs penetration through HE (Table 2). It is worth mentioning the behavior of the Cet cream, which determined the highest permeation of PMA-IONs through HE and, at the same time, the lowest retained amount. These findings might suggest that the larger amount of surfactants used in its preparation, with respect to other tested formulations, influenced the affinity of PMA-IONs for HE, enhancing their permeation. Such evidence was also in agreement with previous published data obtained in vivo using a semi-solid base with a very similar composition [21]. Furthermore, the in vitro penetration results stressed how critical was the physical stability of nanoparticles in the semi-solid matrix for permeating through the skin. Indeed, the permeation of PMA-IONs loaded in HEC was significantly reduced in comparison to other formulations (Table 2). According to the *r*_2_ values, PMA-IONs loaded in both HEC and CMC hydrogel changed their physical state within one week after preparation. Although such changes of their superficial properties were reversible (i.e., adsorption or weak interactions), both polymers caused a reduction of PMA-IONs penetration through human epidermis. Therefore, the selection of a suitable semi-solid vehicle for the nanosystems should be rationalized on the basis of their physical stability at short and long term. The modifications of the physical properties of PMA-IONs loaded in hydrogels and o/w creams were dependent on the formulation features. Thus, the application of a universal methodology to assess the physical stability of these systems could not be considered.

Our results demonstrated that DLS, which is routinely used for the characterization of liquid suspensions, was not the technique of election for semi-solid formulations since the physical status of nanoparticles could be affected by the necessary dilution of the sample. Moreover, DLS was not able to discriminate among particles deriving from the excipients used for the formulation and to be applied to opaque products such as creams due to the signal interferences of the large droplets of the dispersed phase, even if it was used in backscattering mode. To overcome this issue, low-field pulsed NMR, which exploits the magnetic properties of the superparamagnetic core of PMA-IONs, was conveniently proposed as an alternative to directly evidence changes of PMA-IONs dispersed in semi-solid matrices. By the comparison of the two analytical techniques, a wider versatility of low-field pulsed NMR was clearly evidenced since it allowed the direct determination of the physical stability of paramagnetic nanoparticles in opaque vehicles and it did not require any sample dilution. Furthermore, the former technique was more sensitive since it was able to highlight also changes of PMA-IONs physical state that could affect their inability to penetrate the skin (e.g., HEC). As a matter of fact, the reduction of *r*_2_ values in the HEC hydrogel provided a fast evidence of the instability of PMA-IONs, which was in agreement with the permeation data profile.

## Conclusions

The current results support the use of superparamagnetic nanoparticles coated with PMA as a technological platform for drug delivery to human epidermis after topical administration. The loading of PMA-IONs in semi-solid vehicles did not affect their permeation profile only if they maintained their stability over time.

Considering that the physical state of PMA-IONs in semi-solid preparations was the most critical attribute for in vitro permeation performances, the selection of appropriate analytical methods was underlined. In particular, the overall results demonstrated that low-field pulsed NMR allowed to better discriminate the physical stability of superparamagnetic nanosystems in comparison to the conventionally utilized DLS measurements, due to the higher versatility and sensitivity. However, only these techniques provided a deeper insight into a very complex system, allowing to discriminate between irreversible and reversible changes in the physical states of paramagnetic nanosystems. All the tested semi-solid formulations influenced the PMA-IONs penetration through HE; the Cet cream allowed the highest permeation and the lowest retained amount while cold creams and CP hydrogels favored the nanoparticle accumulation into the skin membrane. These results suggest that these two very basic formulations are suitable to be conveniently used to administer PMA-IONs topically in order to improve the efficiency of colloidal nanoparticles in penetrating the skin layers.

## Methods

### Preparation of polymer coated superparamagnetic iron-oxide nanoparticles

PMA-IONs were prepared following previously reported procedures [27–29]. Briefly, iron oxide nanoparticles were synthesized by solvothermal decomposition at high temperature in octadecene from iron oleate precursor resulting in iron oxide core dispersed in chloroform with an average diameter of 12.21 ± 0.8 nm (i.e., IONs). Afterward, 4 mg (0.43 nmols, determined by ICP measurements) of IONs were transferred to water phase by mixing with 136 μL of 0.5 M solution of an amphiphilic polymer [poly-(isobutylene-alt-1-tetradecenemaleic anhydride)], corresponding to 100 monomers. After evaporation under vacuum, 5 mL of pH 12 sodium borate buffer were added to the dried mixture to suspend the nanoparticles (i.e., PMA-IONs). PMA-IONs were twice washed in water. PMA-IONs were purified by electrophoresis gels as described elsewhere [30] and re-suspended in water at a final concentration of 3 mg/mL.

### Preparation of topical semi-solid dosage forms containing PMA-IONs

All the semi-solid formulations were prepared with decreased water content, which was subsequently replaced by the aqueous nanoparticles dispersion at the PMA-IONs at 0.6% w/v concentration after the production. The final loading was fixed at 0.3% w/v. Briefly:

- Hydroxyethyl cellulose (HEC) hydrogel: 2.5% w/w HEC previously wet by 10% w/w glycerol was dispersed in distilled water heated at about 40 °C under magnetic stirring.
- Sodium carboxymethyl cellulose (CMC) hydrogel: 5% w/w CMC previously wet by 10% w/w glycerol was dispersed in distilled water heated at about 60 °C under magnetic stirring.
- Carboxypolymethylene (Carbomer 974P, CP) hydrogel: 0.8% w/w was dispersed in distilled water by a magnetic bar. Then, sodium EDTA and propylene glycol was added. Then, the mixture was neutralized by drop wise addition of a 10% w/v sodium hydroxide solution until the gel formation occurred. Amount of sodium hydroxide was adjusted to pH 7.
- Cetomacrogol (Cet) cream was prepared by heating both the oily (i.e., cetomacrogol 1000, cetostearyl alcohol, liquid paraffin and petrolatum) and aqueous phase at the temperature of about 60 °C. Afterwards, the aqueous phase was added to the oily phase under constant stirring.
- Cold cream was prepared by mixing 4% w/w Sepineo P600, 10% w/w almond oil and water.

In all cases, 0.025% w/v methyl-paraben and 0.075% w/v propyl-paraben were added to water in order to preserve form bacteria and mold contamination.

### Storage stability of PMA-IONs loaded hydrogels and creams

PMA-IONs loaded hydrogels and creams were stored at 25 ± 3 and 40 ± 2 °C over a 2-month period. At predetermined data points, the formulations were visually inspected to evidence aggregate formations. The hydrodynamic diameter, zeta-potential and MRI relaxivity were also measured by DLS.

### Morphology of IONs

The morphology of IONs was detected by TEM analysis, 50 μg/mL of IONs were dispersed in hexane and 50 μg/mL PMA-IONs were dispersed in water. A drop of the resulting solution was placed on a Formvar/carbon-coated copper grid and air-dried. TEM images were obtained by a Tecnai G2 Spirit microscope (Oregon, USA) operating at 120 kV.

### Dynamic light scattering (DLS) and zeta-potential measurements

The mean hydrodynamic diameter of nanoparticle (D_h_), PDI, and zeta-potential (ξ) were measured at 25 °C by DLS method. Aliquots of PMA-IONs suspensions and PMA-IONs-loaded semi-solid formulations were diluted 1:20 in HPLC-grade water, filtered with 0.22 μm nylon filter (VWR, USA) and analyzed by Zetasizer Nano ZS (Malvern Instruments Ltd, UK). According to the National Institute of Standards and Technology, a sample with a PDI < 0.05 is considered monodisperse [31].

### Evaluation of PMA-IONs stability in the semi-solid dosage forms by MRI relaxivity measurement

The study of the variation of the PMA-IONs relaxation time in the semi-solid formulations was made by low-field pulsed NMR Spectrometer mq20 (Bruker The Minispec, Italy). Aliquots 1 mL of were analyzed in a glass vial at 40 °C; a magnetic field was applied and, once the stimulus was interrupted, T_2_ relaxation time was measured. The relaxivity (*r*_2_) of PMA-IONs was calculated according the following equation (Eq. 1):

1 $$r_{2} = \frac{{\left( {\frac{1}{{T_{2} }}} \right)_{sample} - \left( {\frac{1}{{T_{2} }}} \right)_{control} }}{{\left[ {PMA - IONs} \right]}}$$

where (1/T_2_)_sample_ was the inverse of relaxation time of the semisolid matrix containing PMA-IONs, (1/T_2_)_control_ the inverse of relaxation time of the placebo semisolid matrix and the [PMA-IONs] was the concentration of nanoparticles in the semisolid matrix.

All the formulations were analyzed at the nanoparticles concentration of 0.15% w/v. Each analysis was conducted after 0, 2, 20, 32, 41 and 48 days from preparation (n = 3).

### In vitro penetration studies

In vitro permeation and retention studies were performed with the Franz diffusion cells using HE or SC, or a membrane based on regenerated keratin (RKM; 24).

HE was prepared from full-thickness skin following an internal standard procedure [32]. The SC and viable epidermis (VE) were obtained by incubating HE in a Petri dish with 10 mL of 0.005% trypsin in pH 7.4 PBS at 37 ± 1 °C for 18 h. The integrity of HE was checked before the permeation studies by measuring the electrical resistance of HE or SC [33].

The membrane was placed on the Franz diffusion cell whose receptor compartment was filled with degassed 0.9% w/v NaCl solution containing 0.01% w/v NaN_3_ as preservative. The donor compartment was filled with 0.5 mL of aqueous suspension or formulations loaded with 3 mg/mL PMA-IONs. The system was kept at 32 ± 1 °C throughout all the experiment. At experiment end, the receptor phase was withdrawn and analyzed by ICP-OES for determining the iron amount permeated through the membrane after 24 h (Q_24_).

Afterwards, the membrane was gently cleaned to eliminate the unabsorbed PMA-IONs. Subsequently, the sample was dried, weighted and analyzed by ICP-OES to quantify the iron amount retained into the membrane (Q_ret_). The results were expressed as the average of parallel experiments, performed in triplicate. As discussed by Musazzi et al. [33], the Q_ret_/Q_24,HE_ ratio was calculated as parameter to estimate the affinity of PMA-IONs to be retained or to permeate through HE. The higher the Q_ret_/Q_24,HE_, the higher the retention affinity of nanoparticles.

### Nanoparticles quantification by ICP analysis

For the ICP-OES analysis of the samples collected from the Franz diffusion cell receptor chambers, 3 mL of aqua regia were added to the samples and, after 72 h, the samples were diluted with 7 mL of distilled water. All samples were measured in triplicate with Optima 7000 DV ICP-OES (Perkin Elmer, Waltham, USA).

### Epidermis fixation protocol and transmission electron microscopy (TEM) analysis

For the ultrastructural analysis a fragment of each skin specimen was fixed in 2.5% buffered glutaraldehyde, washed with buffer and post-fixed in 1.5% buffered OsO_4_ at 4 °C for 2 h. The specimens were dehydrated in a graded series of alcohol solution and embedded in Epon. The sections were then stained with uranyl acetate and lead citrate and examined in a FEI Tecnai G2 Spirit microscope equipped with a digital camera (Oregon, USA). The obtained sections were deposited on Formvar/carbon-coated copper grid.

### EDX analysis

The STEM image and energy dispersive X-ray analysis (EDX) were performed using a ZEISS LIBRA200FE TEM equipped with an HAADF-STEM (high angular annular dark field scanning electron microscopy) and EDS—Oxford INCA Energy TEM 200. The EDX analysis was performed on the epithelial tissue exposed to the IONs for 24 h and prepared as described in the previous paragraph.

## Additional file

**Additional file 1.** Additional tables and figures.

## Abbreviations

Cet: cetomacrogol
CMC: sodium carboxymethyl cellulose
CP: carboxypolymethylene
D_h_: hydrodynamic diameter of nanoparticle
DLS: dynamic light scattering
HE: human epidermis
HEC: hydroxyethyl cellulose
IONs: iron oxide nanoparticles
o/w: oil in water
PDI: poly dispersity index
PMA-IONs: iron oxide nanoparticles coated by a poly-(isobutylene-*alt*-maleic anhydride)
Q_24_: amount of iron permeated in 24 h
Q_ret_: amount of iron retained in 24 h
*r*_2_: relaxivity
RKM: membrane based on regenerated keratin
SC: stratum corneum
T_2_: relaxation time
VE: viable epidermis
ξ: zeta-potential
