# Supplementary material for: Impact of semi-solid formulations on skin penetration of iron oxide nanoparticles
Source: J Nanobiotechnology. 2017 Feb 17;15:14. doi: 10.1186/s12951-017-0249-6 (PMC5316225; doi:10.1186/s12951-017-0249-6)

**Table S1 -** r_2_-values of PMA-IONs loaded in hydrophilic creams, namely a cold cream (SEP) and cetomacrogol cream (CET), or hydrogel, made of hydroxyethyl cellulose (HEC), sodium carboxymethyl cellulose (CMC) and carbomer 974P (CP), stored at 25 °C.

|  | **Time (days)** | | | | | |
| --- | --- | --- | --- | --- | --- | --- |
|  | **0** | **2** | **20** | **32** | **41** | **48** |
| **SEP** | 289.68±0.00 | 302.23±2.38 | 311.26±0.04 | 310.2±2.58 | 300.31±2.28 | 298.33±2.23 |
| **CET** | 125.96±2.85 | 138.74±8.01 | 144.62±3.22 | 136.95±6.93 | 158.93±3.84 | 124.92±2.89 |
| **HEC** | 977.27±2.25 | 528.55±2.23 | 221.93±0.42 | 197.89±0.18 | 216.45±0.00 | 277.78±0.62 |
| **CMC** | 786.58±4.98 | 395.15±4.47 | 144.55±0.61 | 124.78±1.38 | 114.46±1.42 | 123.13±1.30 |
| **CP** | 676.4±19.29 | 675.94±6.43 | 714.39±4.34 | 754.17±4.45 | 791.38±4.89 | 781.12±6.17 |
| **CONTROL** | 315.74±157.46 | 304.73±12.58 | 389.22±66.88 | 349.16±40.95 | 362.38±46.73 | 318.15±57.63 |

**Table S2.**

Changes in zeta-potential (mV) of PMA-IONs loaded hydrogels during storage at 25 °C (mean value ± St.Dev.; n=3).

| Form. | Storage time (days) | | | | | | | |
| --- | --- | --- | --- | --- | --- | --- | --- | --- |
|  | **0** | **1** | **4** | **8** | **10** | **20** | **30** | **50** |
| HEC | -42.26±1.68 | -39.77±0.59 | -38.43±3.76 | -43.13±4.40 | -39.63±1.55 | -40.70±1.21 | -42.20±2.80 | -39.13±2.10 |
| CMC | -79.70±2.00 | -67.53±3.06 | -69.30±1.13 | -70.63±3.44 | -69.03±3.56 | -67.97±2.15 | -63.00±1.04 | -69.77±0.50 |
| CP | -72.83±5.25 | -74.93±5.40 | -68.50±3.08 | -72.67±3.92 | -73.03±5.88 | -75.20±0.60 | -69.63±1.50 | -70.63±1.60 |

* Blank HEC -9.56±0.28 mV; Blank CMC: -50.93±3.25 mV; Blank CP: -51.90±0.17 mV

**Table S3.**

Changes in D_h_ (and PDI) of PMA-IONs loaded hydrogels during storage at 40 °C (mean value ± St.Dev.; n=3).

| Form. | Storage time (days) | | | | | | |
| --- | --- | --- | --- | --- | --- | --- | --- |
|  | **0** | **2** | **4** | **8** | **20** | **30** | **50** |
| HEC | 29.4±15.3 | 29.7±15.5 | 26.3±1.8 | 34.4±10.4 | 35.7±9.6 | 34.4±12.2 | 42.2±8.6 |
| CMC | 46.6±6.7 | 176.5±26.3 | 318.7±85.2 | n.d. | n.d. | n.d. | n.d. |
| CP | 48.9±20.3 | 43.6±11.5 | 50.5±13.4 | 49.0±5.3 | 57.8±13.5 | 65.5±7.5 | 26.2±6.2 |

n.d. not determined

**Figure S1.**

STEM image of HE tissue with irregular white spots (on the left side) and the relative the EDX spectrum (on the right side). The copper signals come from the TEM grid .


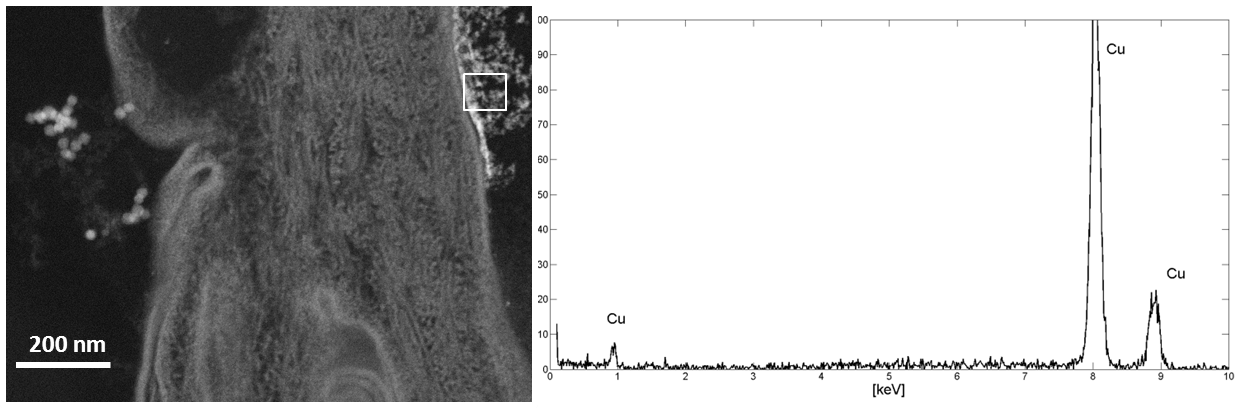

Supplement: Supplementary file 1 — Additional file 1. Additional tables and figures. [file 12951_2017_249_MOESM1_ESM.docx]
